# Dose-Dependent Influences of Ethanol on Ischemic Stroke: Role of Inflammation

**DOI:** 10.3389/fncel.2019.00006

**Published:** 2019-02-12

**Authors:** Guodong Xu, Chun Li, Anne L. Parsiola, Jiyu Li, Kimberly D. McCarter, Runhua Shi, William G. Mayhan, Hong Sun

**Affiliations:** ^1^Department of Cellular Biology & Anatomy, Louisiana State University Health Sciences Center-Shreveport, Shreveport, LA, United States; ^2^Department of Neurology, Hebei General Hospital, Shijiazhuang, China; ^3^Department of Medicine/Feist-Weiller Cancer Center, Louisiana State University Health Sciences Center-Shreveport, Shreveport, LA, United States; ^4^Basic Biomedical Sciences, Sanford School of Medicine, The University of South Dakota, Vermillion, SD, United States

**Keywords:** ethanol, ischemic stroke, adhesion molecule, microglia, neutrophil infiltration, cytokine/chemokine, matrix metalloproteinase

## Abstract

Chronic ethanol consumption dose-dependently affects both incidence and prognosis of ischemic stroke. Our goal was to determine whether the influence of chronic ethanol consumption on ischemic stroke is related to an altered inflammatory profile in the brain. Male C57BL/6J mice were divided into six groups and gavage fed with 0.175, 0.35, 0.7, 1.4, 2.8 g/kg/day ethanol or volume-matched water once a day for 8 weeks. Adhesion molecules, microglial activation, neutrophil infiltration, pro- and anti-inflammatory cytokines/chemokines, blood-brain barrier (BBB) permeability, and matrix metallopeptidases (MMPs) in the cerebral cortex before and following a 90-min unilateral middle cerebral artery occlusion (MCAO)/24-h reperfusion were evaluated. Brain ischemia/reperfusion (I/R) injury was significantly reduced in 0.7 g/kg/day ethanol group (peak blood ethanol concentration: 9 mM) and worsened in 2.8 g/kg/day ethanol group (peak blood ethanol concentration: 37 mM). Baseline E-selectin was downregulated in all ethanol groups, whereas baseline intercellular adhesion molecule-1 (ICAM-1) was only downregulated in 0.35 and 0.7 g/kg/day ethanol groups. Interestingly, baseline vascular cell adhesion molecule-1 (VCAM-1) was upregulated in 0.35, 0.7, and 1.4 g/kg/day ethanol groups. Post-ischemic upregulation of ICAM-1 and E-selectin were suppressed in all ethanol groups. Post-ischemic neutrophil infiltration and microglial activation were significantly less in the low-moderate (0.175–1.4 g/kg/day) ethanol groups but greater in the 2.8 g/kg/day ethanol group compared to the vehicle group. At basal conditions, ethanol increased one pro- and two anti-inflammatory cytokines/chemokines at the 0.7 g/kg/day dose, and 13 pro- and eight anti-inflammatory cytokines/chemokines at the 2.8 g/kg/day dose. After ischemia, 0.7 g/kg/day ethanol suppressed post-ischemic pro-inflammatory cytokines/chemokines and enhanced post-ischemic anti-inflammatory cytokines/chemokines. Moreover, 0.7 g/kg/day ethanol significantly reduced baseline MMP-9 activity and alleviated post-ischemic BBB breakdown. On the other hand, 2.8 g/kg/day ethanol worsened post-ischemic BBB breakdown. Our findings suggest that low-moderate ethanol consumption may prevent ischemic stroke and reduce brain I/R injury by suppressing inflammation, whereas heavy alcohol consumption may induce ischemic stroke and worsen brain I/R injury by aggravating inflammation.

## Introduction

Ischemic stroke continues to be one of the leading causes of mortality and permanent disability worldwide (Favate and Younger, 2016; Benjamin et al., 2017). Intravenous recombinant tissue plasminogen activator (tPA) and endovascular therapy are currently used to treat acute ischemic stroke. Both treatments result in recanalization/reperfusion. Although recanalization/reperfusion is critical for restoring normal function, it paradoxically induces and worsens brain injury, called brain ischemia/reperfusion (I/R) injury (Jean et al., 1998). The mechanisms underlying brain I/R injury are complex and involve several interacting elements, including oxidative/nitrosative stress, activation of apoptotic and autophagic pathways, and increased inflammatory response (Jean et al., 1998; Kalogeris et al., 2012; Chen et al., 2014).

Post-ischemic inflammatory responses are characterized by the accumulation of inflammatory cells and mediators in the ischemic brain. The recruitment of leukocytes (including neutrophils) to the ischemic area appears to be a central feature after transient focal cerebral ischemia (Gronberg et al., 2013). When neutrophils are depleted from the circulation the infarct volume is reduced and cerebral blood flow (CBF) is improved during the reperfusion period (Jean et al., 1998; Herz et al., 2015). The recruitment of leukocytes into the ischemic area is facilitated by upregulation of adhesion molecules on endothelial cells prior to and during reperfusion (Supanc et al., 2011). In addition, the recruitment of leukocytes is associated with inflammatory activation of microglia and subsequent production of matrix metallopeptidases (MMPs), chemokines, and cytokines. Microglia are key modulators of the immune response in the brain. After ischemia, microglia rapidly proliferate, transform their morphology and migrate toward injured neurons (Weinstein et al., 2010). Activated microglia directly contribute to brain I/R injury by phagocytosis and producing inflammatory and cytotoxic mediators (Shukla et al., 2017). MMPs are responsible for remodeling extracellular matrix (Bonnans et al., 2014). In addition to microglia, neurons, astrocytes, and endothelial cells also express MMPs (Turner and Sharp, 2016). After transient focal cerebral ischemia, MMPs are upregulated and activated under the influence of inflammatory mediators (Ceulemans et al., 2010). Activated MMPs stimulate leukocyte adherence and transmigration and promote blood-brain barrier (BBB) breakdown and hemorrhagic transformation (Lakhan et al., 2009; Ceulemans et al., 2010). Cytokines are among the principal mediators of the inflammatory response and are involved in virtually every facet of stroke (Kim et al., 2014). Cytokines can be elaborated by leukocytes, macrophages, endothelial cells and resident cells within the brain (Kim et al., 2014). After transient focal cerebral ischemia, pro-inflammatory cytokines, such as interleukin-1 (IL-1), tumor necrosis factor-α (TNF-α), and IL-6, stimulate and aggravate the inflammatory response (Shukla et al., 2017).

Ethanol is one of the most commonly and regularly used chemical substances. Epidemiological studies suggest that low-moderate ethanol intake lowers the incidence of ischemic stroke and reduces mortality and infarct volume from ischemic stroke, whereas heavy ethanol consumption increases the incidence of ischemic stroke and worsens the prognosis of ischemic stroke (Hansagi et al., 1995; Ikehara et al., 2008; Patra et al., 2010; Ronksley et al., 2011; Ducroquet et al., 2013; Zhang et al., 2014). Ischemic stroke is most often caused by atherosclerosis, which has been well recognized as a chronic inflammatory process (Bruno et al., 2008). The relationship between regular ethanol intake and incidence of atherosclerosis appears to be U-shaped (Kiechl et al., 1998). Thus, our goal was to determine whether the influence of ethanol on ischemic stroke is related to an altered inflammatory profile in the brain.

## Materials and Methods

### Animal Models of Ethanol Preconditioning

This study was carried out in accordance with the recommendations of the National Institutes of Health Guide for the Care and Use Laboratory Animals. The protocol was approved by the Institutional Animal Care and Use Committee at the Louisiana State University Health Science Center-Shreveport. One hundred twenty male C57BL/6J mice (20–25 g) were randomly divided into six groups: vehicle (*n* = 24), 0.175 g/kg/day ethanol (*n* = 16), 0.35 g/kg/day ethanol (*n* = 16), 0.7 g/kg/day ethanol (*n* = 24), 1.4 g/kg/day ethanol (*n* = 16), and 2.8 g/kg/day ethanol (*n* = 24). Ethanol groups were gavage fed with 10 ml/kg 1.75% (0.175 g/kg/day ethanol group), 3.5% (0.35 g/kg/day ethanol group), 7% (0.7 g/kg/day ethanol group), 14% (1.4 g/kg/day ethanol group) or 28% (2.8 g/kg/day ethanol group) ethanol once a day for 8 weeks. The vehicle group was gavage fed with 10 ml/kg water. Fasting blood glucose was measured by Bayer Breeze2 Blood Glucose Meter (Bayer HealthCare, Mishawaka, IN, USA). Prior to the measurement, mice were fasted for 12 h during the daytime. To determine whether 8-week feeding changes the peak concentration of blood ethanol, time courses of plasma ethanol concentration in the 0.7 and 2.8 g/kg/day groups were measured using an Ethanol Assay Kit (ab65343, Abcam) at the beginning and end of 8-week feeding period. The measurement was performed according to the manufacturer’s instructions. Same mice were used for same time point in each group. Blood pressure and heart rate were measured using a CODA mouse tail-cuff system (Kent Scientific, Torrington, CT, USA). Prior to the actual measurement, mice were trained for three consecutive days to acclimate to being restrained and to also having the tail cuff placed on them. At the end of 8 weeks of feeding, all mice were subjected to transient focal cerebral ischemia.

### Transient Focal Cerebral Ischemia

To avoid a possible effect of acute ethanol, alcohol was not given on the day before the experiment. Transient focal cerebral ischemia was induced by unilateral middle cerebral artery occlusion (MCAO). Since disability-free outcome is better when reperfusion is established less than 90 min after the onset of ischemic stroke (Meretoja et al., 2014), 90-min was selected as the MCAO period. Prior to the procedure, mice were anesthetized with isoflurane (induction at 5% and maintenance at 1.5%) in a gas mixture containing 30% O_2_/70% N_2_ *via* a facemask. Body temperature was maintained with a temperature controlled heating pad (Harvard Apparatus, March, Germany). A laser-Doppler flow probe (PERIMED, PF 5010 LDPM Unit, Sweden) was attached to the right side of the dorsal surface of the skull to monitor regional CBF (rCBF). The right common and external carotid arteries were exposed and ligated. The MCA was occluded by inserting a silicon rubber-coated monofilament (Doccol Corporation, Sharon, MA, USA) from the basal part of the external carotid artery and advanced cranially into the internal carotid artery to the point where the MCA branched off from the internal artery. The onset of the MCAO was indicated by an immediate drop in rCBF. After the occlusion of the right MCA for 90 min, reperfusion was achieved by withdrawing the suture and reopening the common carotid artery. Animals were allowed to recover for 24 h.

### Assessment of Neurological Deficits, Infarct Volume and Edema

A 24-point scoring system was used to evaluate sensorimotor deficits at 24 h of reperfusion (Sun et al., 2008). Sensorimotor testing was graded on a scale of 0–3 each on spontaneous activity, symmetry of movement, response to vibrissae touch, floor walking, beam walking, symmetry of forelimbs, climbing wall of wire cage, reaction to touch on either side of trunk. Neurological scores were assigned as follows: 0, complete deficit; 1, definite deficit with some function; 2, decreased response or mild deficit; 3, no evidence of deficit/symmetrical responses. After neurological evaluation, 30 mice (*n* = 5 for each group) were euthanized and exsanguinated. The brains were quickly removed and placed in ice-cold saline for 5 min, and cut into six 1.75 mm-thick coronal sections. Sections were stained with 2% 2,3,5-triphenyltetrazolium chloride (TTC; Sigma) for 15 min at 37°C. Sliced images were digitalized, the volumes of infarct lesion, ipsilateral hemisphere and contralateral hemisphere were measured using ImageJ. Total infarct volume was expressed as a percentage of the contralateral hemisphere. Edema was determined by the volume ratio of the ipsilateral hemisphere to the contralateral hemisphere and expressed as a percentage increase of the contralateral hemisphere. All researchers involved in assessing brain injury were blinded to the experimental groups.

### Western Blot Analysis

Thirty mice (*n* = 5 for each group) were euthanized and exsanguinated. Cortical tissues were isolated from the peri-infarct area and contralateral corresponding area to measure protein expression of intercellular adhesion molecule-1 (ICAM-1), vascular cell adhesion molecule-1 (VCAM-1), E-selectin and P-selectin. The brains were cut into six 1.75 mm-thick coronal sections. Under the microscope, the infarct core was identified as an opaque area, and the area bordering 2 mm the infarct core was considered as the peri-infarct area (Choi et al., 2015; McCarter et al., 2017). A researcher who was blinded to the experimental groups collected cortical tissues from the peri-infarct area. The samples were homogenized in ice-cold lysis buffer (150 mmol/l NaCl, 50 mmol/l Tris-HCl, 10 mmol/l EDTA, 0.1% Tween-20, 1% Triton, 0.1% mercaptoethanol, 0.1 mmol/l phenylmethyl sulfonyl fluorides, 5 μg/ml leupeptin, and 5 μg/ml aprotinin, pH 7.4). Lysates were then centrifuged at 12,000× *g* for 20 min at 4°C, and the supernatants were collected. Protein concentration of the supernatants was determined by the Bradford protein assay (Bio-Rad). SDS-PAGE was performed on a 10% gel on which 20 μg of total protein per well was loaded. After SDS-PAGE, the proteins were transferred to a polyvinylidene difluoride membrane. Immunoblotting was performed using mouse anti-ICAM-1 (sc-8439, Santa Cruz Biotechnology), rabbit anti-VCAM-1 (ab134047, Abcam), rabbit anti-E-selectin (ab18981, Abcam) and goat anti-P-selectin (AF737, R&D systems) as primary antibodies and peroxidase conjugated goat anti-mouse, mouse anti-rabbit, and mouse anti-goat IgG as the secondary antibody. The bound antibody was detected using enhanced chemiluminescence (ECL) detection (Genesee Scientific for ICAM-1, VCAM-1; Thermo Scientific for E-selectin, P-selectin), and the bands were analyzed using ChemiDoc^TM^ MP Imaging System (Bio-Rad). To quantify, protein expression of ICAM-1, VCAM-1, E-selectin and P-selectin was normalized to GAPDH and expressed as percentage changes to the vehicle without I/R.

### Immunohistochemistry Staining

Thirty mice (*n* = 5 for each group) were anesthetized and perfused transcardially with 1× phosphate-buffered saline (PBS), followed by 4% paraformaldehyde in 0.1 mmol/L PBS. The brains were removed, fixed overnight in 4% paraformaldehyde in 0.1 M PBS, dehydrated in a graded series of sugar solutions over the course of 72 h, then embedded in O.C.T. compound (Fisher Scientific) and quick frozen for 5 min in liquid nitrogen. The frozen brains were then cut into 14 μm sections and placed on frost-free slides. The sections were washed with 1× PBS, blocked with 10% bovine serum albumin (BSA) for at least 1 h, and then incubated overnight at 4°C with 1:100 rabbit anti-myeloperoxidase (anti-MPO; Abcam) for visualization of neutrophils or 1:100 rabbit anti-ionized calcium binding adaptor molecule 1 (anti-Iba1; Wako Chemicals Inc., Richmond, VA, USA) for visualization of microglia as primary antibodies. The sections were then incubated with 1:200 AlexaFluor 555 donkey anti-rabbit (Santa Cruz Technology) for 1 h at room temperature. Sections were mounted with DAPI mounting medium with Vector shield and visualized using a fluorescence microscope. Cells positive for MPO represented infiltrating neutrophils. For quantitative analysis, positive cells were counted in three separate areas per section surrounding the infarct area in at least three slides per mouse in each group. To quantify microglia, cells positive for Iba1 were observed and counted. Resting microglia present with long processes extending from their cell body. Resting microglia present with long processes extending from their cell body. Upon activation, microglia change form from highly ramified to completely lacking processes. In addition, activated microglia show increased Iba1 expression (Taylor and Sansing, 2013; Yang et al., 2015). Thus, microglia with increased Iba1 expression and three processes or less were deemed to be activated (Ahmad et al., 2014; Yang et al., 2015).

### Cytokine Array

Expression of cytokines in the right cerebral hemisphere was measured using the Proteome Profiler Mouse Cytokine Array Kit, Panel A (ARY006, R&D Systems) in 24 mice (*n* = 4 for vehicle, vehicle + I/R, 0.7 g/kg/day ethanol, 0.7 g/kg/day ethanol + I/R, 2.8 g/kg/day ethanol, and 2.8 g/kg/day ethanol + I/R). The array was performed according to the manufacturer’s instructions. Results were analyzed using ImageJ software and expressed as percentage change relative to vehicle without I/R.

### IgG Staining

Three sections (Bregma 1.21 mm, −0.23 mm and −1.31 mm) per mouse (*n* = 5 for each group) were blocked with 10% BSA for 1 h. Subsequently, the sections were incubated overnight at 4°C with 1:200 goat anti-mouse IgG (BA-9200, Vector), followed by incubating with 1:200 AlexaFluor 555 (Invitrogen) for 1 h at room temperature. Staining was analyzed with a fluorescence microscope and an image analysis system (NIS-Elements, Nikon). The mean fluorescence intensity of equal size around the infarct core and contralateral corresponding area was measured. IgG staining was normalized to contralateral corresponding area and expressed as percentage changes to the vehicle group.

### Gelatin Zymography

MMP activity in the cortical tissues prepared for Western Blot analysis was measured using gelatin zymography. Gelatin zymography was carried out using SDS-polyacrylamide gels containing 0.8% gelatin (Sigma, St. Louis, MO, USA). After electrophoresis, the gels were washed with a buffer (pH 7.5) containing 50 mM Tris-HCl, 5 mM CaCl_2_, 1 μM ZnCl_2_, 0.02% NaN_3_, and 2.5% Triton X-100 to remove the SDS and renature the gelatinases. Gels were then developed in a buffer (pH 7.5) containing 50 mM Tris-HCl, 5 mM CaCl_2_, 1 μM ZnCl_2_, 0.02% NaN_3_ for 7 days at 37°C. Enzymatic activity was visualized as negative staining with 0.25% Coomassie Brilliant Blue R-250 (Sigma, St. Louis, MO, USA). The molecular sizes of gelatinolytic activity were determined by comparison to Molecular Weight Markers (Bio-Rad, Hercules, CA, USA). The gel was photographed and analyzed using ChemiDoc^TM^ MP Imaging System (Bio-Rad).

### Statistical Analysis

Data are presented as means ± SE. Differences in physiological parameters, cerebral I/R injury, microglia activation, neutrophil infiltration, brain edema, and IgG staining between groups were evaluated by one-way analysis of variance (ANOVA) followed by Dunnett’s test for multiple comparisons to the control group. Differences in protein expression of adhesion molecules, cytokines and chemokines, and MMP activity between groups were evaluated by two-way ANOVA followed by Bonferroni’s test for multiple within and between group comparisons. All analyses were done using statistical analysis software SAS9.4 for windows (SAS Institute, Cary, NC, USA). *P* ≤ 0.05 was considered as statistical significant.

## Results

### Physiological Parameters

After 8-weeks of gavage feeding there was no significant difference in body weight among six groups (Table 1). Since cerebral I/R injury was significantly altered in the 0.7 g/kg/day and 2.8 g/kg/day ethanol groups, mean arterial blood pressure (MABP), heart rate, and fasting blood glucose were measured in the vehicle, 0.7 g/kg/day, and 2.8 g/kg/day ethanol groups. There was no significant difference in MABP, heart rate, or fasting blood glucose among three groups (Table 1).

**Table 1:** Effect of ethanol consumption on body weight, blood pressure, heart rate and fasting blood glucose.

	Vehicle	0.175 g/kg/day	0.35 g/kg/day	0.7 g/kg/day	1.4 g/kg/day	2.8 g/kg/day
Body weight (g)	29.4 ± 0.4	28.6 ± 0.6	27.7 ± 0.6	29.1 ± 0.3	27.9 ± 0.5	28.7 ± 0.5
MABP (mmHg)	81.0 ± 3.8	ND	ND	82.3 ± 2.8	ND	90.6 ± 4.5
Heart rate (bpm)	637 ± 40	ND	ND	564 ± 57	ND	644 ± 17
Fasting blood glucose (mg/dl)	139 ± 8	ND	ND	118 ± 12	ND	156 ± 10

*Values are means ± SE for five rats in each group. ND stands for Not Determined*.

### Cerebral I/R Injury

**Figure 1:**
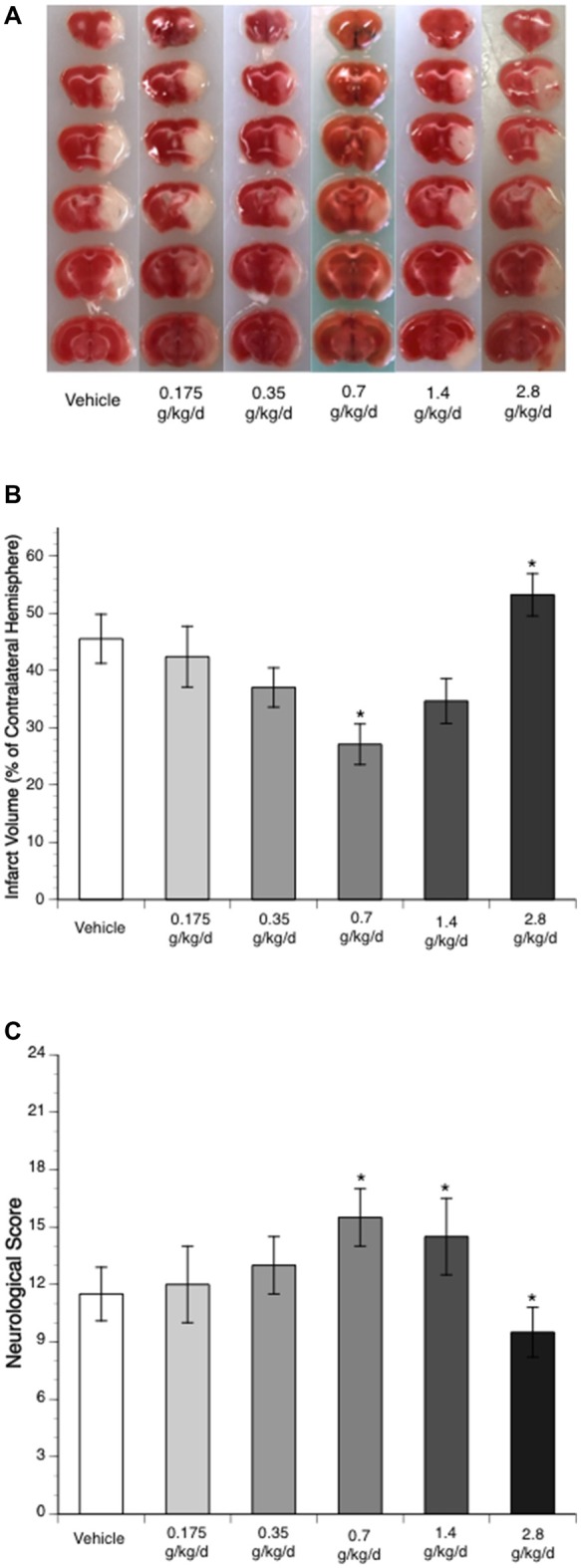
Influence of ethanol on brain injury following a 90-min middle cerebral artery occlusion (MCAO)/24-h reperfusion. **(A)** Representative brain sections stained with 2,3,5-triphenyltetrazolium chloride (TTC). **(B)** Total infarct volume (*n* = 6). **(C)** Neurological score (*n* = 15). Values are means ± SE. *P < 0.05 vs. Vehicle.

**Figure 2:**
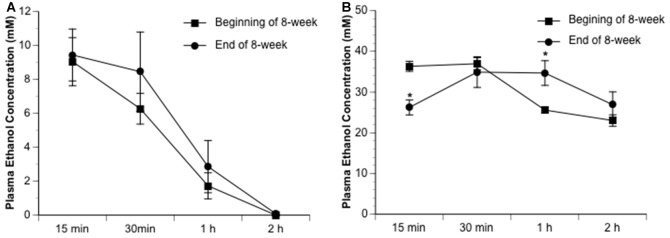
Dynamic change of plasma ethanol concentration in the 0.7 g/kg/day **(A)** and 2.8 g/kg/day **(B)** ethanol groups at the beginning and end of an 8-week feeding period. Values are means ± SE for four mice in each group. *P < 0.05 vs. Beginning of 8-week.

Ethanol altered the total infarct volume at 24 h of reperfusion as indicated by a significant main effect of gavage feeding (*F*_(5,27)_ = 8.9; *p* = 0.000043, *n* = 6). *Post hoc* analysis revealed that 0.7 g/kg/day ethanol reduced infarct volume compared to vehicle (*p* = 0.010214; Figures 1A,B). On the other hand, 2.8 g/kg/day ethanol increased infarct volume compared to vehicle (*p* = 0.036302; Figure 1B). Consistently, ethanol altered the neurological deficits at 24 h of reperfusion as indicated by a significant main effect of gavage feeding (*F*_(5,84)_ = 10.92; *p* < 0.000001, *n* = 15). *Post hoc* analysis showed that 0.7 g/kg/day ethanol significantly improved neurological deficits (*p* = 0.001570), whereas 2.8 g/kg/day ethanol significantly worsened neurological deficits compared to vehicle (*p* = 0.041920; Figure 1C). Interestingly, although the total infarct volume was not significantly reduced (*p* = 0.193254), neurological deficits significantly improved in the 1.4 g/kg/day ethanol group (*p* = 0.006; Figure 1C). Since both infarct volume and neurological deficits were significantly altered in the 0.7 g/kg/day and 2.8 g/kg/day ethanol groups, plasma ethanol concentration was measured at 15 min, 30 min, 1 h and 2 h after gavage feeding in these two groups. At the beginning of an 8-week feeding period, the peak concentrations (0.7 g/kg/day ethanol group: 9.0 ± 0.4 mM; 2.8 g/kg/day ethanol group: 37.0 ± 1.5 mM) appeared at 15 min in the 0.7 g/kg/day ethanol group and 30 min in the 2.8 g/kg/day ethanol group (Figure 2). The ethanol concentration reduced rapidly and approached to zero at 2 h in the 0.7 g/kg/day ethanol group. In contrast, plasma ethanol concentration declined slowly in the 2.8 g/kg/day ethanol group. Eight-week feeding produced a significant decrease and a significant increase in plasma ethanol concentration at 15 min (*p* = 0.013) and 1 h (*p* = 0.049), respectively, in the 2.8 g/kg/day ethanol group. However, 8-week feeding did not affect the peak concentration and its time point in both the 0.7 g/kg/day and 2.8 g/kg/day ethanol groups (Figure 2).

### Protein Expression of Adhesion Molecules

**Figure 3:**
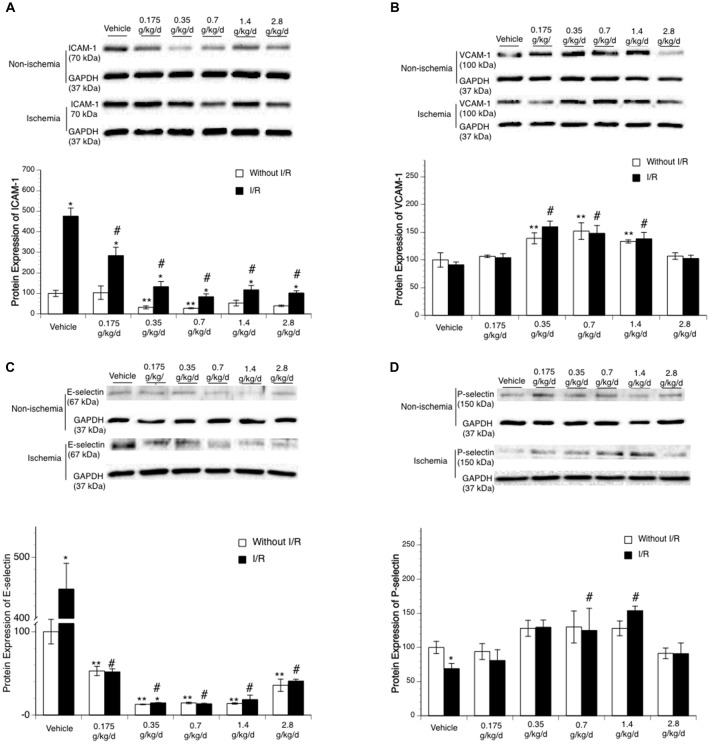
Influence of ethanol on expression of intercellular adhesion molecule-1 (ICAM-1; **A**), vascular cell adhesion molecule-1 (VCAM-1; **B**), E-selectin **(C)** and P-selectin **(D)** following a 90-min MCAO/24-h reperfusion. Values are means ± SE for five mice in each group. Data shown are representative blots for each group. Ischemic side and contralateral side were run on separate gels with two samples from the vehicle group as internal control. **P < 0.05 vs. Vehicle without ischemia/reperfusion (I/R). *P < 0.05 vs. Without I/R. ^#^*P* < 0.05 vs. Vehicle with I/R.

Ethanol altered baseline expression of ICAM-1 as indicated by a significant main effect of gavage feeding (*F*_(5,24)_ = 4.48; *p* = 0.005020, *n* = 5). *Post hoc* analysis showed that 0.35 and 0.7 g/kg/day ethanol significantly downregulated baseline ICAM-1 (*p* = 0.031723 and *p* = 0.020119, respectively; Figure 3A). A 90-min MCAO significantly upregulated ICAM-1 at 24 h of reperfusion in all groups (Vehicle group: *F*_(1,8)_ = 81.90; *p* = 0.000018, *n* = 5; 0.175 g/kg/day group: *F*_(1,8)_ = 12.23; *p* = 0.008117, *n* = 5; 0.35 g/kg/day group: *F*_(1,8)_ = 14.85; *p* = 0.004856, *n* = 5; 0.7 g/kg/day group: *F*_(1,8)_ = 15.75; *p* = 0.004127, *n* = 5; 1.4 g/kg/day group: *F*_(1,8)_ = 6.77; *p* = 0.031492, *n* = 5; 2.8 g/kg/day group: *F*_(1,8)_ = 31.23; *p* = 0.000517, *n* = 5; Figure 3A). There was a significant interaction of ethanol and ischemic stroke on ICAM-1 expression at 24 h of reperfusion (*F*_(11,48)_ = 15.53; *p* < 0.000001, *n* = 5). Mice fed with ethanol had significantly less upregulation in ICAM-1 at 24 h of reperfusion (*F*_(5,24)_ = 31.54; *p* < 0.000001, *n* = 5). *Post hoc* analysis showed that 0.175, 0.35, 0.7, 1.4, and 2.8 g/kg/day ethanol significantly reduced post-ischemic ICAM-1 (*p* = 0.000223, *p* < 0.000001, *p* < 0.000001, *p* < 0.000001, and *p* < 0.000001, respectively; Figure 3A). In addition to ICAM-1, ethanol also reduced baseline expression of E-selectin as indicated by a significant main effect of gavage feeding (*F*_(5,24)_ = 32.53; *p* < 0.000001, *n* = 5). *Post hoc* analysis showed that 0.175, 0.35, 0.7, 1.4, and 2.8 g/kg/day ethanol significantly downregulated baseline E-selectin (*p* < 0.00001, *p* < 0.000001, *p* = 0.000163, *p* < 0.000001, and *p* < 0.000001, respectively; Figure 3C). A 90-min MCAO significantly upregulated E-selectin at 24 h of reperfusion in the vehicle group (*F*_(1,8)_ = 65.21; *p* = 0.000041, *n* = 5). There was a significant interaction of ethanol and ischemic stroke on E-selectin expression (*F*_(11,48)_ = 62.58; *p* < 0.000001, *n* = 5). Mice fed with ethanol had significantly less upregulation in E-selectin at 24 h of reperfusion (*F*_(5,24)_ = 100.17; *p* < 0.000001, *n* = 5). *Post hoc* analysis revealed that only 0.35 g/kg/day ethanol significantly upregulated E-selectin at 24 h of reperfusion (*F*_(1,8)_ = 10.10; *p* = 0.013023, *n* = 5; Figure 3C). In contrast to ICAM-1 and E-selectin, ethanol upregulated baseline expression of VCAM-1 (*F*_(5,24)_ = 23.20; *p* < 0.000001, *n* = 5). *Post hoc* analysis showed that 0.35, 0.7, and 1.4 g/kg/day ethanol significantly upregulated baseline VCAM-1 (*p* = 0.000045, *p* < 0.000001, and *p* = 0.005964, respectively; Figure 3B). Ethanol did not significantly alter baseline expression of P-selectin (Figure 3D). In addition, 90-min MCAO did not significantly upregulate either VCAM-1 or P-selectin at 24 h of reperfusion in any group. Interestingly, P-selectin was slightly but significantly downregulated at 24 h of reperfusion in the vehicle group (*F*_(1,8)_ = 6.46; *p* = 0.034622, *n* = 5; Figure 3D). Ethanol altered post-ischemic VCAM-1 (*F*_(5,24)_ = 11.84; *p* = 0.000008, *n* = 5) and P-selectin (*F*_(5,24)_ = 5.16; *p* = 0.002354, *n* = 5). *Post hoc* analysis revealed that 0.35, 0.7, and 1.4 g/kg/day ethanol significantly increased post-ischemic VCAM-1 (*p* = 0.000171, *p* = 0.000028, and *p* = 0.010020, respectively; Figure 3B). *Post hoc* analysis also revealed that 0.35 and 0.7 g/kg/day ethanol significantly increased post-ischemic P-selectin (*p* = 0.001953 and *p* = 0.022819, respectively; Figure 3D). However, there was no significant interaction of ethanol and ischemic stroke on either VCAM-1 (*F*_(11,48)_ = 0.40; *p* = 0.846695, *n* = 5) or P-selectin (*F*_(11,48)_ = 0.89; *p* = 0.495995, *n* = 5).

### Microglial Activation

**Figure 4:**
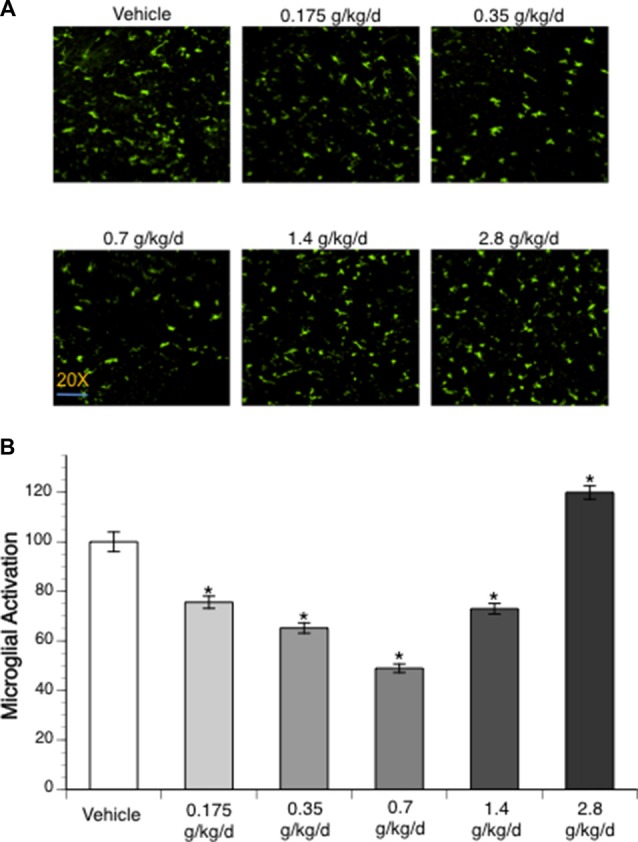
Influence of ethanol on microglial activation following a 90-min MCAO/24-h reperfusion. **(A)** Representative lba1 staining. **(B)** Values are mean ± SE for five mice in each group. *P < 0.05 vs. Vehicle.

To assess microglial activation, immunofluorescence staining with Iba1 was analyzed. No activated microglia were detected at 24 h of reperfusion in the contralateral hemisphere of ischemic brain. Ethanol altered post-ischemic microglial activation at 24 h of reperfusion in the ipsilateral hemisphere of the ischemic brain as indicated by a significant main effect of gavage feeding (*F*_(5,56)_ = 52.23; *p* < 0.000001, *n* = 5). *Post hoc* analysis showed that 0.175, 0.35, 0.7, and 1.4 g/kg/day ethanol significantly inhibited post-ischemic microglial activation (*p* = 0.00004, *p* < 0.000001, *p* < 0.000001 and *p* = 0.000001, respectively; Figures 4A,B). In contrast, 2.8 g/kg/day ethanol significantly promoted post-ischemic microglial activation compared to vehicle (*p* = 0.000613; Figures 4A,B).

### Neutrophil Infiltration

**Figure 5:**
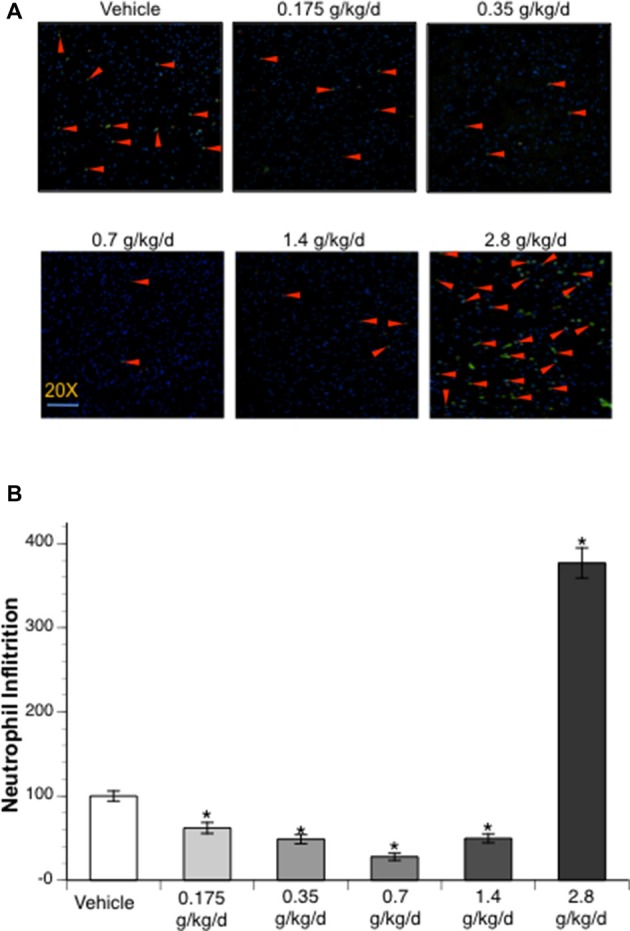
Influence of ethanol on neutrophil infiltration following a 90-min MCAO/24-h reperfusion. **(A)** Representative MPO staining. **(B)** Values are mean ± SE for five mice in each group. *P < 0.05 vs. Vehicle.

Immunofluorescence staining with MPO was counted to examine neutrophil infiltration. No neutrophil infiltration was detected at 24 h of reperfusion in the contralateral hemisphere. Ethanol altered post-ischemic neutrophil infiltration at 24 h of reperfusion in the ipsilateral hemisphere of the ischemic brain as indicated by a significant main effect of gavage feeding (*F*_(5,52)_ = 53.92; *p* < 0.000001, *n* = 5). *Post hoc* analysis revealed that 0.175, 0.35, 0.7, and 1.4 g/kg/day ethanol significantly attenuated post-ischemic neutrophil infiltration (*p* = 0.022635, *p* = 0.000818, *p* = 0.000002 and *p* = 0.001079, respectively; Figures 5A,B). On the other hand, 2.8 g/kg/day ethanol significantly exacerbated post-ischemic neutrophil infiltration compared to vehicle (*p* < 0.000001; Figures 5A,B).

### Cytokines and Chemokines

**Figure 6:**
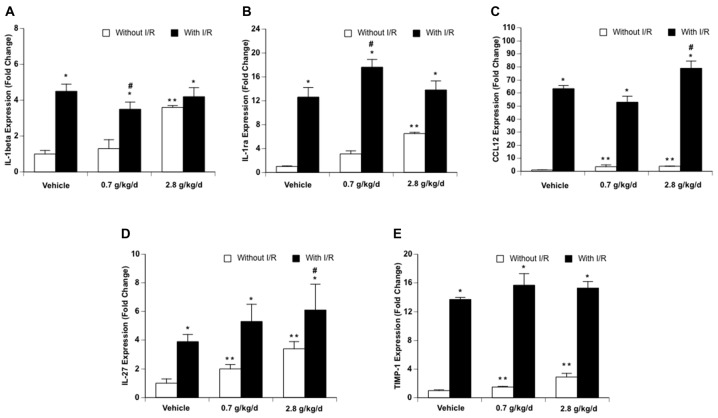
Influence of ethanol on expression of interleukin-1β (IL-1β; **A**), IL-1ra **(B)**, CCL-12 **(C)**, IL-27 **(D)** and TIMP-1 **(E)** following a 90-min MCAO/24-h reperfusion. Values are means ± SE for four mice in each group. **P < 0.05 vs. Vehicle without I/R. *P < 0.05 vs. Without I/R. ^#^*P* < 0.05 vs. Vehicle with I/R.

Since cerebral I/R injury was significantly altered in the 0.7 g/kg/day and 2.8 g/kg/day ethanol groups, cytokines and chemokines were only measured in the vehicle, 0.7 g/kg/day, and 2.8 g/kg/day ethanol groups. Thirty-three cytokines/chemokines were detected using the cytokine array (Figure 6 and Table 2). Ethanol altered 13 baseline pro-inflammatory cytokines/chemokines (IL-1β, IL-3, IL-7, IL-17, IL-23, sICAM, TREM-1, CCL3, CCL12, CXCL2, CXCL9, CXCL10, CXCL11) as indicated by significant main effects of gavage feeding (IL-1β: *F*_(2,9)_ = 99.16; *p* < 0.000001, *n* = 4; IL-3: *F*_(2,9)_ = 194.83; *p* < 0.000001, *n* = 4; IL-7: *F*_(2,9)_ = 31.09; *p* = 0.000091, *n* = 4; IL-17: *F*_(2,9)_ = 8.77; *p* = 0.007697, *n* = 4; IL-23: *F*_(2,9)_ = 4.15; *p* = 0.052952, *n* = 4; sICAM: *F*_(2,9)_ = 4.19; *p* = 0.051810, *n* = 4; TREM-1: *F*_(2,9)_ = 4.91; *p* = 0.036217, *n* = 4; CCL3: *F*_(2,9)_ = 20.07; *p* = 0.000481, *n* = 4; CCL12: *F*_(2,9)_ = 15.38; *p* = 0.001249, *n* = 4; CXCL2: *F*_(2,9)_ = 5.37; *p* = 0.029185, *n* = 4; CXCL9: *F*_(2,9)_ = 13.43; *p* = 0.001988, *n* = 4; CXCL10: *F*_(2,9)_ = 4.79; *p* = 0.038350, *n* = 4; CXCL11: *F*_(2,9)_ = 6.41; *p* = 0.018620, *n* = 4). *Post hoc* analysis revealed that 0.7 g/kg/day ethanol only significantly increased baseline CCL12 (*p* = 0.006008; Figure 6C), whereas 2.8 g/kg/day ethanol significantly increased baseline IL-1β (*p* < 0.000001), IL-3 (*p* < 0.000001), IL-7 (*p* = 0.000116), IL-17 (*p* = 0.010393), IL-23 (*p* = 0.037578), sICAM (*p* = 0.036577), TREM-1 (*p* = 0.026932), CCL3 (*p* = 0.000472), CCL12 (*p* = 0.000965), CXCL2 (*p* = 0.024882), CXCL9 (*p* = 0.001251), CXCL10 (*p* = 0.038815), and CXCL11 (*p* = 0.013630) compare to vehicle (Figure 6 and Table 2). On the other hand, ethanol altered all eight detected anti-inflammatory cytokines/chemokines (IL-1ra, IL-4, IL-13, IL-27, G-CSF, M-CSF, TIMP-1, and CXCL13) as indicated by significant main effects of gavage feeding (IL-1ra: *F*_(2,9)_ = 21.21; *p* = 0.000393, *n* = 4; IL-4: *F*_(2,9)_ = 4.97; *p* = 0.035154, *n* = 4; IL-13: *F*_(2,9)_ = 14.60; *p* = 0.001494, *n* = 4; IL-27: *F*_(2,9)_ = 43.65; *p* = 0.000023, *n* = 4; G-CSF: *F*_(2,9)_ = 41.27; *p* = 0.000029, *n* = 4; M-CSF: *F*_(2,9)_ = 5.93; *p* = 0.022740, *n* = 4; TIMP-1: *F*_(2,9)_ = 65.65; *p* = 0.000004, *n* = 4; CXCL13: *F*_(2,9)_ = 31.68; *p* = 0.000084, *n* = 4). *Post hoc* analysis revealed that 0.7 g/kg/day ethanol significantly increased baseline IL-27 (*p* = 0.010523) and TIMP-1 (*p* = 0.039746; Figure 6C), whereas 2.8 g/kg/day ethanol significantly increased baseline IL-1ra (*p* = 0.000234), IL-4 (*p* = 0.023458), IL-13 (*p* = 0.001525), IL-27 (*p* = 0.000013), G-CSF (*p* = 0.000026), M-CSF (*p* = 0.021383), TIMP-1 (*p* = 0.019979) and CXCL13 (*p* = 0.000056) compare to vehicle (Figure 6 and Table 2). A 90-min MCAO increased nearly all pro- [IL-1α (*p* = 0.004446), IL-1β (*p* = 0.000049), IL-3 (*p* = 0.003363), IL-6 (*p* = 0.000427), IL-7 (*p* = 0.001322), IL-16 (*p* = 0.006295), IL-17 (*p* = 0.002063), IL-23 (*p* = 0.008776), TNFα (*p* = 0.008415), sICAM (*p* = 0.005868), C5a (*p* = 0.002926), TREM-1 (*p* = 0.000090), IFNγ (*p* = 0.015976), CCL2 (*p* < 0.000001), CCL3 (*p* = 0.000165), CCL4 (*p* = 0.000002), CCL5 (*p* = 0.000333), CCL12 (*p* < 0.000001), CXCL1 (*p* < 0.000001), CXCL2 (*p* = 0.000002), CXCL9 (*p* = 0.001983), CXCL10 (*p* = 0.003656), CXCL11 (*p* = 0.017108), CXCL12 (*p* = 0.003795), and CXCL13 (*p* = 0.004357)] and anti-(IL-1ra (*p* = 0.000022), IL-4 (*p* = 0.006545), IL-13 (*p* = 0.006153), IL-27 (*p* < 0.000001), G-CSF (*p* = 0.000538), M-CSF (*p* = 0.004005), TIMP-1 (*p* < 0.000001) and CXCL13 (*p* = 0.000056) inflammatory cytokines/chemokines at 24 h of reperfusion in the vehicle group. The number of upregulated pro- and anti-inflammatory cytokines/chemokines at 24 h of reperfusion was less in two ethanol groups. Twelve pro-[IL-1β (*p* = 0.000325), IL-3 (*p* = 0.016041), IL-6 (*p* = 0.012197), IL-16 (*p* = 0.022580), IL-17 (*p* = 0.042719), C5a (*p* = 0.010869), TREM-1 (*p* = 0.003024), CCL2 (*p* = 0.000093), CCL3 (*p* = 0.000472), CCL12 (*p* < 0.000001), CXCL1 (*p* = 0.000006), and CXCL2 (*p* = 0.001563)] and four anti-[IL-1ra (*p* = 0.000008), IL-27 (*p* = 0.000154), G-CSF (*p* = 0.008262), and TIMP-1 (*p* = 0.000185)] inflammatory cytokines/chemokines were increased in the 0.7 g/kg/day ethanol group. Only ten pro- [IL-1β (*p* = 0.003581), IL-3 (*p* = 0.009448), IL-6 (*p* = 0.005024), TREM-1 (*p* = 0.008930), CCL2 (*p* = 0.000002), CCL3 (*p* = 0.040100), CCL4 (*p* = 0.002640), CCL12 (*p* < 0.000001), CXCL1 (*p* = 0.000001), and CXCL2 (*p* = 0.000067)] and three anti- [IL-1ra (*p* = 0.013861), IL-27 (*p* = 0.02689), and TIMP-1 (*p* = 0.000008)] inflammatory cytokines/chemokines were increased in the 2.8 g/kg/day ethanol group (Figure 6 and Table 2). There were significant interactions of ethanol and ischemic stroke on IL-1β (*F*_(5,18)_ = 25.71; *p* = 0.000005, *n* = 4), IL-3 (*F*_(5,18)_ = 6.00; *p* = 0.010051, *n* = 4), CCL12 (*F*_(5,18)_ = 30.40; *p* = 0.000002, *n* = 4), IL-1a (*F*_(5,18)_ = 5.86; *p* = 0.010941, *n* = 4). *Post hoc* analysis revealed that 0.7 g/kg/day ethanol significantly reduced post-ischemic IL-1β (*p* = 0.020488) and increased IL-1ra (*p* = 0.047886) at 24 h of reperfusion. *Post hoc* analysis also revealed that 2.8 g/kg/day ethanol significantly increased post-ischemic IL-3 (*p* = 0.006474), IL-23 (*p* = 0.034201), and CCL12 (*p* = 0.000689) at 24 h of reperfusion (Figure 6 and Table 2).

**Table 2:** Effect of ethanol consumption on cytokines and chemokines in the cerebrum before and following a 90-min middle cerebral artery occlusion (MCAO)/24-h reperfusion.

Cytokines/Chemokines	Vehicle	0.7 g/kg/day	2.8 g/kg/day	Vehicle + I/R	0.7 g/kg/day + I/R	2.8 g/kg/day + I/R
IL-1α	1.0 ± 0.1	1.0 ± 0.2	1.1 ± 0.3	1.8 ± 0.2*	1.6 ± 0.3	1.4 ± 0.5
IL-2	1.0 ± 0.2	2.1 ± 0.5	2.3 ± 0.4	1.2 ± 0.1	1.8 ± 1.2	4.1 ± 2.0
IL-3	1.0 ± 0.2	1.1 ± 0.2	5.1 ± 0.2**	6.0 ± 1.1*	4.0 ± 0.9*	17.8 ± 3.4*^#^
IL-4	1.0 ± 0.3	2.4 ± 1.1	4.5 ± 1.0**	3.2 ± 0.5*	2.6 ± 0.9	9.8 ± 2.6^#^
IL-6	ND	ND	ND	1.0 ± 0.1*	1.2 ± 0.3*	0.4 ± 0.1*
IL-7	1.0 ± 0.1	1.2 ± 0.4	3.5 ± 0.2**	2.9 ± 0.3*	2.6 ± 0.7	3.4 ± 0.5
IL-13	1.0 ± 0.1	1.2 ± 0.1	2.5 ± 0.4**	2.0 ± 0.2*	1.7 ± 0.3	1.5 ± 0.8
IL-16	1.0 ± 0.1	1.1 ± 0.2	1.1 ± 0.3	1.8 ± 0.2*	2.1 ± 0.3*	0.8 ± 0.5
IL-17	1.0 ± 0.3	1.0 ± 0.5	3.4 ± 0.5**	2.8 ± 0.2*	2.8 ± 0.4*	2.1 ± 0.5
IL-23	1.0 ± 0.2	2.4 ± 1.1	4.5 ± 1.0**	3.2 ± 0.5*	2.6 ± 0.9	9.8 ± 2.6^#^
TNFα	1.0 ± 0.6	2.4 ± 1.6	4.7 ± 0.5	12.2 ± 2.8*	16.9 ± 6.9	11.4 ± 6.9
G-CSF	1.0 ± 0.4	1.6 ± 0.4	4.6 ± 0.1**	4.9 ± 0.5*	6.3 ± 1.2*	4.7 ± 1.8
M-CSF	1.0 ± 0.2	1.2 ± 0.3	2.4 ± 0.4**	2.2 ± 0.2*	3.5 ± 1.5	4.5 ± 1.0
sICAM	1.0 ± 0.0	1.3 ± 0.2	1.5 ± 0.2**	1.2 ± 0.1*	1.3 ± 0.1	1.5 ± 0.1
C5a	1.0 ± 0.2	1.5 ± 0.2	1.4 ± 0.4	4.0 ± 0.6*	4.0 ± 0.7*	3.2 ± 0.9
TREM-1	1.0 ± 0.1	1.8 ± 1.1	3.6 ± 0.1**	12.3 ± 1.2*	10.4 ± 1.5*	14.2 ± 2.8*
IFN-γ	1.0 ± 0.2	0.9 ± 0.2	1.5 ± 0.3	2.7 ± 0.4*	2.6 ± 0.9	3.0 ± 1.0
CCL2	1.0 ± 0.1	0.8 ± 0.5	1.5 ± 0.1	8.8 ± 0.1*	8.9 ± 0.7*	7.9 ± 0.3*
CCL3	1.0 ± 0.4	1.9 ± 1.1	6.7 ± 0.2**	8.6 ± 0.8*	9.2 ± 1.0*	10.5 ± 1.5*
CCL4	ND	ND	ND	1.2 ± 0.1*	1.5 ± 0.6	1.4 ± 0.3*
CCL5	1.0 ± 0.4	2.7 ± 1.1	3.8 ± 0.4	6.8 ± 0.7*	5.1 ± 1.8	5.5 ± 1.3
CXCL1	1.0 ± 0.3	0.8 ± 0.2	1.1 ± 0.3	15.8 ± 0.3*	16.1 ± 1.0*	14.1 ± 0.6*
CXCL2	1.0 ± 0.1	1.4 ± 0.1	3.0 ± 0.8**	18.7 ± 1.0*	19.0 ± 3.2*	19.0 ± 1.4*
CXCL9	1.0 ± 0.2	1.6 ± 0.4	2.7 ± 0.1**	3.4 ± 0.4*	3.4 ± 0.9	2.7 ± 0.9
CXCL10	1.0 ± 0.5	4.7 ± 1.1	5.2 ± 1.4**	3.9 ± 0.3*	3.1 ± 1.0	3.4 ± 1.2
CXCL11	1.0 ± 0.1	2.0 ± 0.8	4.4 ± 0.9**	3.1 ± 0.6*	2.5 ± 0.9	2.6 ± 1.9
CXCL12	1.0 ± 0.1	2.2 ± 0.8	2.6 ± 0.5	1.4 ± 0.1*	1.4 ± 0.3	1.5 ± 0.3
CXCL13	1.0 ± 0.3	3.0 ± 0.3	7.5 ± 0.9**	2.5 ± 0.2*	3.2 ± 1.4	6.4 ± 1.0^#^

Values are means ± SE for four mice in each group. ND stands for Not Detectable. **P < 0.05 vs. Vehicle. *P < 0.05 vs. Without ischemia/reperfusion (I/R). ^#^P < 0.05 vs. Vehicle + I/R.

### BBB Breakdown

**Figure 7:**
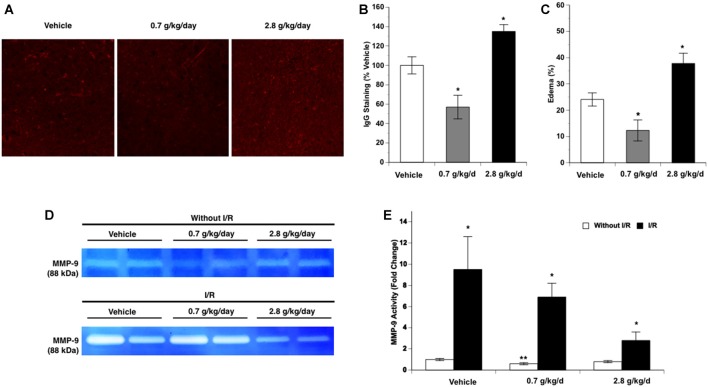
Influence of ethanol on blood-brain barrier (BBB) permeability following a 90-min MCAO/24-h reperfusion. **(A)** Representative IgG staining. **(B)** Values are means ± SE for five mice in each group. **(C)** Values are means ± SE for six mice in each group. **(D)** Representative gelatin zymography. **(E)** Values are means ± SE for five mice in each group. *P < 0.05 vs. Vehicle. **P < 0.05 vs. Vehicle without I/R. ^#^*P* < 0.05 vs. Without I/R.

Again, since cerebral I/R injury was significantly altered in the 0.7 g/kg/day and 2.8 g/kg/day ethanol groups, BBB breakdown-related measurements were only conducted in the vehicle, 0.7 g/kg/day, and 2.8 g/kg/day ethanol groups. No IgG leakage was detected at 24 h of reperfusion in the contralateral hemisphere. Ethanol altered post-ischemic IgG leakage at 24 h of reperfusion in the ipsilateral hemisphere of the ischemic brain as indicated by a significant main effect of gavage feeding (*F*_(2,12)_ = 16.43; *p* = 0.000366, *n* = 5). *Post hoc* analysis showed that 0.7 g/kg/day ethanol reduced post-ischemic IgG leakage compared to vehicle (*p* = 0.015192; Figure 7B). In contrast, 2.8 g/kg/day ethanol worsened post-ischemic IgG leakage compared to vehicle (*p* = 0.044803; Figures 7A,B). Ethanol also altered post-ischemic brain edema in the ipsilateral hemisphere of the ischemic brain as indicated by a significant main effect of gavage feeding (*F*_(2,15)_ = 15.45; *p* = 0.000229, *n* = 5). *Post hoc* analysis revealed that 0.7 g/kg/day ethanol significantly alleviated post-ischemic brain edema (*p* = 0.036270), whereas 2.8 g/kg/day significantly worsened the post-ischemic brain edema (*p* = 0.018484; Figure 7C). Ethanol altered baseline MMP-9 activity as indicated by a significant main effect of gavage feeding (*F*_(2,12)_ = 6.26; *p* = 0.013741, *n* = 5). *Post hoc* analysis showed that 0.7 g/kg/day ethanol significantly reduced baseline MMP-9 activity (*p* = 0.010701; Figures 7D,E). A 90-min MCAO significantly increased MMP-9 activity at 24 h of reperfusion in all three groups (Vehicle group: *F*_(1,8)_ = 81.90; *p* = 0.000018, *n* = 5; 0.7 g/kg/day group: *F*_(1,8)_ = 20.84; *p* = 0.001837, *n* = 5; 2.8 g/kg/day group: *F*_(1,8)_ = 7.15; *p* = 0.028171, *n* = 5; Figures 7D,E). There was no significant interaction of ethanol and ischemic stroke on MMP-9 activity at 24 h of reperfusion (*F*_(5,24)_ = 3.14; *p* = 0.061657, *n* = 5). In addition, ethanol did not significantly alter MMP-9 activity at 24 h of reperfusion (*F*_(2,12)_ = 3.14; *p* < 0.079986, *n* = 5; Figures 7D,E). In contrast to MMP-9, MMP-2 activity was not altered by ethanol and at 24 h of reperfusion (data not shown).

## Discussion

The present study investigated the influence of chronic consumption of low to high doses of ethanol on basal and post-ischemic inflammatory profiles in the brain. There are several new findings from this study. First, 0.35–0.7 g/kg/day ethanol tends to reduce baseline expression of ICAM-1 and E-selectin, but increase baseline expression of  VCAM-1. Second, post-ischemic upregulation of ICAM-1 and E-selectin were attenuated in all ethanol groups. Third, low to moderate ethanol alleviated but high-dose ethanol aggravated post-ischemic microglial activation and neutrophil infiltration. Fourth, ethanol tended to dose-dependently increase both pro- and anti-inflammatory cytokines/chemokines at basal conditions. Fifth, low-dose ethanol reduced post-ischemic pro-inflammatory cytokines/chemokines and enhanced post-ischemic anti-inflammatory cytokines/chemokines. Sixth, low-dose ethanol reduced but high-dose ethanol worsened post-ischemic BBB breakdown. Thus, ethanol may affect both basal and post-ischemic inflammatory profiles of the brain in a dose-dependent manner. Low-dose ethanol tends to induce an anti-inflammatory effect, whereas high-dose ethanol produces an inflammatory effect.

Although numerous experimental studies have investigated the effect of ethanol consumption on cardiovascular diseases, there have only been a limited amount of studies investigating the effect of ethanol consumption on ischemic brain damage. In an early study, Mandybur and Mendenhall (1983) found that chronic alcoholism significantly contributes to the risk of mortality associated with ischemic brain infarction in gerbils. Later, Favalli et al. (2002) found that chronic ethanol consumption leads to an increased excitotoxicity and ischemic brain damage during early withdrawal in rats. Recently, Oliveira et al. (2014) found that heavy ethanol consumption exacerbates post-ischemic motor impairment and cortical neuronal loss in female adolescent rats. Our previous studies have shown that 2-month feeding with a liquid diet containing high-dose ethanol (6.4% v/v) worsened post-ischemic brain damage in rats (Sun et al., 2008; Zhao et al., 2010), whereas the diet containing low-dose ethanol (1% v/v) protected the brain against its I/R injury in both rats and mice (Zhao et al., 2011; Sun et al., 2012). To mimic the drinking pattern of humans, different doses of ethanol were given once a day *via* oral gavage feeding in the present study. We found that 0.7 g/kg/day ethanol significantly protected against brain I/R injury and 2.8 g/kg/day ethanol significantly exacerbated brain I/R injury. The peak blood ethanol concentration of the dose associated with a neuroprotective effect was 9.0 mM, which usually can be seen in a man with average body weight (70 kg or 154 Ibs) after ingestion of one and a half American standard drinks (14 grams of ethanol/each; Fisher et al., 1987). On the other hand, the peak blood ethanol concentration of the dose associated with a detrimental effect was 37.0 mM, which usually can be seen in a man with average body weight after ingestion of slightly more than seven American standard drinks. Therefore, the results of the present study complement and extend that which we have reported previously.

As far as we are aware, the present study is the first to systematically investigate the influence of chronic consumption of low to high doses of ethanol on early post-ischemic inflammation in the brain. We found that transient focal cerebral ischemia increased the expression of ICAM-1 and E-selectin, but not VCAM-1 and P-selectin at 24 h of reperfusion. Interestingly, the magnitude of the increase in ICAM-1 and E-selectin was significantly less in all ethanol groups when compared to the vehicle. The selectins facilitate the diapedesis of leukocytes on the endothelial surface while the immunoglobulin superfamily mediates the firm adhesion and transendothelial migration of leukocytes (Langer and Chavakis, 2009). Animals deficient in ICAM-1 or treated with strategies that block ICAM-1 have decreased ischemic damage and less brain neutrophil infiltration (Kitagawa et al., 1998). In addition, E-selectin inhibition is associated with improved neurological outcome (Huang et al., 2000). Of the various types of leukocytes, neutrophils are among the first to infiltrate the ischemic brain (Gronberg et al., 2013). Neutrophils may worsen brain I/R injury by obstructing capillaries resulting in reduced blood flow during reperfusion as well as by releasing cytotoxic products. Numerous studies have shown that inhibition of neutrophil infiltration is associated with a decrease in brain I/R injury (Egashira et al., 2013; Herz et al., 2015). In the present study, neutrophil infiltration was significantly inhibited in the 0.175–1.4 g/kg/day ethanol groups. The maximum inhibition was observed in the 0.7 g/kg/day ethanol group, in which the brain I/R injury was significantly reduced. Therefore, the neuroprotective effect of low-dose ethanol may be related to a reduction in expression of adhesion molecules and subsequent neutrophil infiltration.

Microglia are resident immune cells of the brain and are important modulators of homeostasis and immune response in the brain (Ziebell et al., 2015). Upon stimulation, microglia become activated and undergo several key morphological changes characterized by an amoeboid shape with little to no extending processes (Ziebell et al., 2015). Activated microglia may contribute to brain I/R injury *via* phagocytosis and elaboration of neuroinflammatory mediators toxic to cells (Ceulemans et al., 2010). In the present study, although post-ischemic microglia activation was significantly attenuated in the 0.175–1.4 g/kg/day ethanol groups, the maximum reduction occurred in the 0.7 g/kg/day ethanol group. Therefore, the neuroprotective effect of low-dose ethanol may be linked to a reduction in microglial activation.

Following transient focal cerebral ischemia, cytokines/chemokines are elaborated from injured neurons, infiltrated leukocytes, activated astrocytes, microglia, and endothelial cells (Kim et al., 2014). Pro-inflammatory cytokines, such as IL-1β, TNFα, and IL-6, contribute to brain I/R injury by stimulating pro-apoptotic signaling and cytotoxic proteins, activating microglia, and increasing the expression of adhesion molecules (Doll et al., 2014). In contrast, anti-inflammatory cytokines, such as IL-1ra, IL-4, and IL-10, inhibit post-ischemic inflammation by inhibiting pro-inflammatory cytokines and suppressing cytokine receptor expression and downstream signaling (Kim et al., 2014). Chemokines play a crucial role in the infiltration of leukocytes under inflammatory conditions (Kim et al., 2014). In the present study, one pro-inflammatory mediator, IL-1β, was less and one anti-inflammatory cytokine, IL-1ra, was greater in the 0.7 g/kg/day ethanol group compared to the vehicle group. IL-1 is a strong neurotoxic mediator and has two isoforms, IL-1α and IL-1β. However, IL-1β rather than IL-1α is considered to be more engaged in ischemic brain damage (Boutin et al., 2001). IL-1ra is an endogenous inhibitor of IL-1. Overexpression of IL-1ra as well as treatment with IL-1ra led to reduced ischemic brain damage (Mulcahy et al., 2003). Thus, it is conceivable that the neuroprotective effect of low-dose ethanol may also be related to an altered post-ischemic expression of cytokines/chemokines.

In the present study, post-ischemic inflammatory response produced conflicting results in the 2.8 g/kg/day ethanol group where the cerebral I/R injury was significantly exacerbated. Surprisingly, although neutrophil infiltration and microglial activation were increased, post-ischemic upregulation in adhesion molecules was reduced. Furthermore, both pro-inflammatory and anti-inflammatory cytokines/chemokines were increased after ischemic stroke. In addition to adhesion molecules, pro-inflammatory cytokines/chemokines and BBB breakdown can also influence the infiltration of neutrophils (Kim et al., 2014). In the present study, post-ischemic increase in three pro-inflammatory (IL-3, IL-23, and CCL12) was significant greater in the 2.8 g/kg/day ethanol group. Unfortunately, no studies that we are aware of have attempted to relate alterations of IL-3 and CCL12 to neutrophil infiltration following ischemic stroke. CCL12 is a potent monocyte chemokine homologous to human CCL2 (Sarafi et al., 1997). Although the role of CCL12 in ischemic brain damage has not been elucidated, overexpression of CCL2 worsens ischemic brain damage (Chen et al., 2003). A recent study report that IL-23 can aggravate neuron damage and further impair the integrity of BBB (Wang et al., 2015). In the present study, post-ischemic BBB breakdown was significantly worsened in the 2.8 g/kg/day ethanol group. On the other hand, reactive oxygen species (ROS) and extracellular ATP from dying cells have been demonstrated to promote post-ischemic microglial activation (Doll et al., 2014). We previously have found that heavy ethanol consumption exacerbates brain I/R injury by increasing NMDA-mediated excitotoxicity and NAD(P)H oxidase-mediated oxidative stress (Zhao et al., 2010, 2011). Thus, the increased neutrophil infiltration and microglia activation may be resulted from exacerbated cell death and BBB breakdown. Post-ischemic inflammation begins immediately after arterial occlusion and contributes to potential enlargement of the infarct size. In the present study, although we were not able to directly establish the correlation between post-ischemic inflammatory response and infarct volume, we evaluated the inflammatory response in the peri-infarct area at 24 h of reperfusion. Recent studies found that the healing type of activated microglia was predominated in the ischemic core while the pre-inflammatory type of activated microglia was predominated in the peri-infarct area (Denes et al., 2007; Villarreal et al., 2016). The spatial distribution of the microglia phenotypes suggests that the peri-infarct area at early stage may represent the enlargement of injured and damaged brain tissue over time.

In the present study, ethanol did not alter baseline P-selectin, but upregulated baseline VCAM-1 and downregulated baseline ICAM-1 and E-selectin at low-moderate doses. All these adhesion molecules have been extensively linked to atherosclerosis (Galkina and Ley, 2007). Thus, the net effect of low-dose ethanol on these adhesion molecules cannot be estimated. However, it has been reported that combined deficiency of E-selectin and P-selectin could produce the strongest inhibitory effects on atherosclerosis (Galkina and Ley, 2007). In the present study, although low-dose ethanol slightly increased baseline CCL-12, it significantly activated anti-inflammatory mediators, IL-27 and TIMP-1. In addition, low-dose ethanol reduced baseline activity of MMP-9. IL-27 has been previously shown to inhibit atherosclerosis (Hirase et al., 2013). MMP-9 not only contributes to atherosclerotic plaque progression but also associates with cap rupture (Vacek et al., 2015). TIMP-1 is a tissue inhibitor of metalloproteinases. Thus, low-dose ethanol consumption may induce an anti-inflammatory property rather than an inflammatory effect under basal conditions. The relationship between regular ethanol intake and incidence of atherosclerosis appears to be U-shaped (Kiechl et al., 1998). It is possible that the beneficial effect of low-dose ethanol against atherosclerosis as well as subsequent ischemic stroke is related to its anti-inflammatory propensity. In contrast to low-dose ethanol, while high-dose ethanol also activated the anti-inflammatory system, it increased most detected pro-inflammatory cytokines/chemokines, suggesting a propensity towards vascular inflammation.

## Conclusion

The present study is the first to systematically evaluate the influence of chronic ethanol consumption on the inflammatory profile in the brain under basal conditions and after transient focal cerebral ischemia. We found that the inflammatory profile during low-moderate ethanol consumption tends to prevent ischemic stroke and reduce brain I/R injury. On the other hand, inflammation may contribute to the increased ischemic stroke and exacerbated brain I/R injury during heavy ethanol consumption. Therefore, anti-inflammatory therapeutic strategies may be able to significantly improve prognosis of ischemic stroke in heavy alcohol users but not in low-moderate alcohol users.

## Author Contributions

HS and WM conceived the experiments. GX, CL, AP, JL, KM and HS conducted the experiments. GX, CL, RS (Biostatistician), and HS analyzed the results. All authors reviewed the manuscript.

## Conflict of Interest Statement

The authors declare that the research was conducted in the absence of any commercial or financial relationships that could be construed as a potential conflict of interest.
